# Triple burden of malnutrition among Vietnamese 0·5–11-year-old children in 2020–2021: results of SEANUTS II Vietnam

**DOI:** 10.1017/S1368980024001186

**Published:** 2024-05-24

**Authors:** Nga Thuy Tran, Van Khanh Tran, Duong Thanh Tran, Tu Tran Ngoc Nguyen, Son Duy Nguyen, Ha Thu Nguyen, Tu Song Nguyen, Tung Van Thanh Le, Phuong Thi Lan Nguyen, Hanh Thi Dang, Hoa Anh Le, Gerard Wong, Ilse Khouw

**Affiliations:** 1 National Institute of Nutrition, Hanoi, Vietnam; 2 FrieslandCampina, Amersfoort, The Netherlands

**Keywords:** Malnutrition, Vitamin and mineral deficiencies, Dietary intake, Children, Vietnam, SEANUTS II

## Abstract

**Objective:**

SEANUTS II Vietnam aims to obtain an in-depth understanding of the nutritional status and nutrient intake of children between 0·5 and 11·9 years old.

**Design:**

Cross-sectional survey.

**Setting:**

A multistage cluster systematic random sampling method was implemented in different regions in Vietnam: North Mountainous, Central Highlands, Red River Delta, North Central and Coastal Area, Southeast and Mekong River Delta.

**Participants:**

4001 children between 6 months and 11·9 years of age.

**Results:**

The prevalence of stunting and underweight was higher in rural than in urban children, whereas overweight and obese rates were higher in urban areas. 12·0 % of the children had anaemia and especially children 0·5–1 year old were affected (38·6 %). Low serum retinol was found in 6·2 % of children ≥ 4 years old. The prevalence of vitamin D insufficiency was 31·1 % while 60·8 % had low serum Zn. For nutrient intake, overall, 80·1 % of the children did not meet the estimated energy requirements. For Ca intake, ∼60 % of the younger children did not meet the RNI while it was 92·6 % in children >7 years old. For vitamin D intake, 95·0 % of the children did not meet recommended nutrient intakes.

**Conclusions:**

SEANUTS II Vietnam indicated that overnutrition was more prevalent than undernutrition in urban areas, while undernutrition was found more in rural areas. The high prevalence of low serum Zn, vitamin D insufficiency and the inadequate intakes of Ca and vitamin D are of concern. Nutrition strategies for Vietnamese children should consider three sides of malnutrition and focus on approaches for the prevention of malnutrition.

## Graphical abstract

**Figure:**
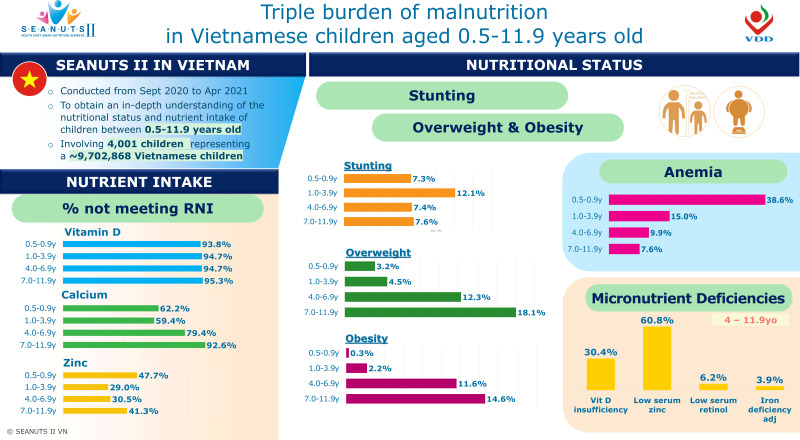

Over the past few decades, the Asian low-middle-income countries had strong socio-economic development, which has both positive and negative effects on almost every aspect of human life, including the change of lifestyle and eating habits^(1)^ with a transitional trend towards a more diverse yet not more healthy diet^(2,3)^, especially in urban areas and among children^(4)^. Childhood malnutrition and undernourishment can lead to a number of health outcomes that can negatively impact children’s life trajectories^(5)^. Poor nutrition status can lead to disadvantageous health conditions in later stages of life such as disabilities, cognitive impairment in children, poor school performance, increased risk of long-term chronic illness, reduced adult income and negative impact on national GDP and productivity^(6–8)^

Vietnam has been undergoing a period of rapid development, which is marked by the transition of diet from a poor and simple ration to a varied one, rich in animal protein and lipids and processed food^(2,9,10)^. This nutrition transition relates to the social, cultural and economic changes in the context of demographic transition. A consequence of this transition is plausibly the co-existence of undernourishment and food insecurity as well as emerging problems such as overweight/obesity and non-communicable diseases^(11,12)^.

The past decades have seen these issues in nutrition transition garnering attention by many researchers, socio-economic experts and policy makers. According to a survey conducted by the National Institute of Nutrition (NIN) in 2010^(13)^, although underweight and stunting among children under 5 have decreased relatively fast and continuously, Vietnam is still among the 36 countries which have high stunting rates on a global scale^(10)^. Le Danh Tuyen et al.^(14)^ conducted a data analysis study based on the results of three studies: the 2000 General Nutrition Survey^(9)^, the 2010 Nutrition Census^(13)^ and the 2011 Southeast Asian Nutrition Surveys (SEANUTS) Vietnam^(11)^. They concluded that there were both child undernourishment and overweight/obese problems at the same time, which was different per region, with high rates of undernourishment in the rural areas and overweight/obesity prevalent in urban areas^(14)^. In addition to the dual burden of malnutrition, studies have also shown that micronutrient deficiencies remain a public health problem in children under 6 years of age and women of reproductive age^(12)^.

Improving nutritional status and dietary intake among children has been priorities for the Vietnamese government since 2000 (2001–2010 National Nutrition Strategy^(15)^, 2011–2020 National Nutrition Strategy^(10)^) and have continuously been among the objectives of the 2021–2030 National Nutrition Strategy with special attention to controlling stunting, overweight/ obesity and micronutrient deficiencies^(16)^. Progress on health and nutrition indicators that are recommended by WHO, UNICEF and FAO should be closely monitored to timely propose early interventions with an effective prevention strategy. Vietnam’s latest General Nutrition Survey was implemented in 2019–2020. However, aspects such as dietary intake and vitamin B_12_ and D status in children were missing. Therefore, this SEANUTS II project was conducted, as a follow-up study of the first SEANUTS (2011)^(11)^, to provide a comprehensive insight into the nutritional issues that children in Vietnam face today.

The present study will provide information on the nutritional status, anaemia, Fe, Zn, vitamin A, vitamin D and vitamin B_12_ status and dietary intakes of children representative for national level and urban/rural for the development of a comprehensive national plan of actions for malnutrition interventions.

## Methodology

The Nutrition Survey of Vietnamese children is part of the South East Asian Nutrition Surveys II (SEANUTS II), which was a multicentre study carried out on 13 933 children aged 0·5–12·9 years in Vietnam, Indonesia, Malaysia and Thailand. A detailed description of the design of SEANUTS II can be found elsewhere (Tan S et al, South East Asian Nutrition Surveys (SEANUTS) II – a multi-country evaluation of nutrition and lifestyle indicators in children aged 12 years and below: Rationale and design, submitted).

In Vietnam, the cross-sectional SEANUTS II survey was conducted from September 2020 to April 2021 and used a multistage cluster systematic random sampling method to select participants from four regions, i.e. North mountainous and Central Highlands, Red River Delta, North Central and Coastal and Southeast and Mekong River Delta Vietnam. The sampling was based on the 2019 national population data by the General Statistics Office, Ministry of Planning and Investment of Vietnam^(17)^. In the first stage, one city and two rural provinces were selected in each region and considered as primary sampling units. The study population was recruited from four big cities and eight rural provinces (twelve cities/provinces in total). In the second stage, in each primary sampling unit, three community-based clusters and three school-based clusters were selected by probability proportional to size and considered as secondary sampling units. In the third stage, children were randomly selected from the children list in each community-based cluster (total of thirty-six communes), and children in primary schools were randomly selected from the children list in each school (total of thirty-six schools). The inclusion criteria included apparently healthy Vietnamese children aged between 0·5 and 11·9 years with parental signed informed consent, without physical disability nor genetic disorders. Children who were ill or were absent on the day of measurements were excluded from the study. The sample was then weighted to reflect the distribution of sex, age and area of residence in the general population within Vietnam^(17)^. Subjects were grouped into four age groups, (0·5–0·9 years), (1·0–3·9 years), (4·0–6·9 years) and (7·0–11·9 years) to represent the life pattern changes in infants and children.

Data collection for children aged less than 6 years was done via commune health centres and that for children 6–11·9 years was done in primary schools. Assessments included anthropometric measurements, food consumption and 24-h recall, and from a subsample blood was collected for biochemical analysis. Nurses, anthropometrists, interviewers and technicians were trained by NIN. Training and assessment methods were standardised and harmonised via standard operating procedures for all countries involved. Health staff at the provincial, district and commune level were trained in all aspects regarding the study and given clear and detailed explanations about their roles and responsibilities in the study.

### Sample size estimation

The sample size estimation was based on the prevalence of stunting, overweight and obesity and the prevalence of anaemia, Fe deficiency, low serum retinol, low serum Zn and vitamin D deficiency as reported from the General Nutrition Survey (GNS) in 2010^(13)^ and SEANUTS I^(11)^. The following formula was used:

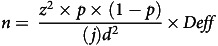




where *n* is the sample size, *z* defines the level of confidence required: *z* = 1·96 for a 95 % confidence level; *p* is an estimate of the key indicator(s) to be measured by the survey in the population of interest; *d* is the desired level of precision and *j* is the expected response rate. The largest sample size for the respective key indicator was used. The required sample size of 4088 subjects covered a design effect of 2·0 and a consent agreement loss of 25 %.

### Anthropometric measurements

Body weight and height were measured in all the children. Weight was measured with a SECA 874 digital weighing scale to the nearest 0·1 kg. Length in children aged < 2 years was measured in the supine position with SECA 417 infantometer to the nearest 0·1 cm. Height was measured in the standing position for children aged from 2 years with a SECA 217 stadiometer to the nearest 0·1 cm. The instruments were calibrated daily. The sd scores (z-scores) of weight, height/ length and BMI were derived using the age- and sex-specific WHO growth references for 0–4 years (WHO 2006^(18)^ using the software WHO Anthro version 3.2.2 and for 5–11 years; WHO 2007^(19)^ using the WHO AnthroPlus version 1.0.4 software). Anthropometric status was assessed using the following indicators: weight-for-age z-scores (WAZ) < –2 for underweight (for < 5 years old), weight-for-height z-scores (WHZ) < –2 for wasting (for < 5 years old), height-for-age z-scores (HAZ) < –2 for stunting; BMI-for-age z-scores (BAZ) < –2 for thinness (for 5–11 years old), BAZ > 2 to ≤ 3 for overweight and > 3 for obesity in children < 5 years old and BAZ > 1 to ≤ 2 for overweight and > 2 for obesity in children 5–11 years old. Children with implausible Z-score values were excluded, when WAZ < –5 or WAZ > 5, HAZ < –6 or HAZ > 6, WHZ < –6 or WHZ > 5, or BAZ < –5 or BAZ > 5.

### Haematological and biochemical indicators

For children from 0·5 to 3·9 years old (*n* 444), fingerpick was conducted to measure Hb by HemoCue (HemoCue Angholm). Early-morning venous blood samples for children aged from 4 to 11·9 years (*n* 1054) were obtained for haematological and biochemical screening tests following a 12-h overnight fast. Trained phlebotomist performed venapuncture to obtain a maximum of 8 ml whole blood in a trace-element free vacutainer with clot activated tube (Vacuette, Greiner Bio One) and 2 ml whole blood in EDTA tube following the blood collection protocols, storage and analysis standard operation procedure (SOP). Primary school children were taken 8 ml of venous blood, and children from 4 to 6 years old were taken 5 ml of venous blood including 3 ml whole blood and 2 ml EDTA blood. Immediately after blood sample collection, the Hb concentration was measured from whole blood using the HemoCue device. Whole blood was centrifuged at 3000 g for 10 min at room temperature. The supernatant plasma was aliquoted into 500 ml pre-labelled Eppendorf tubes and was subsequently kept frozen at –20°C until transported (on dry ice) to the laboratory of the NIN where they were stored at –80°C until analysis. Samples were analysed at the end of the study at NIN laboratory. Serum samples were analysed for ferritin, C-reactive protein (CRP), alpha-1 glycoprotein (AGP), retinol, 25-hydroxyvitamin D, vitamin B_12_ and Zn concentrations. Serum ferritin and CRP concentrations were measured by Immunoturbidimetric method using Beckman Coulter, Inc., USA (Cliniqa Corporation). The concentrations of AGP, used as a marker of inflammation and infection, were measured by ELISA using commercial kits (Mybiosource) with quality controls obtained from Bio-Rad Laboratories (Liquicheck Immunology Control, Bio-Rad Laboratories). Serum retinol concentration was determined by reverse-phase LC/MS/MS (Sciex Qtrap 6500+). Serum 25-hydroxyvitamin D concentration^(20)^ and vitamin B_12_^(21)^ were determined by LC/MS/MS (Sciex Qtrap 6500+ and Sciex Qtrap 5500, respectively) with quality controls approved by CDC, US. Zn concentration was analysed using a flame atomic absorption spectrophotometer (GBC, Avanta+) using trace element-free procedures and powder free gloves (Latex Surgical Glove), and results were verified using reference materials (Liquicheck, Bio-Rad Laboratories). The within-assay CV for serum ferritin, Zn, retinol, 25-hydroxyvitamin D, vitamin B_12_, CRP and AGP ranged from 2·8 to 6·8 %, and between-assay variability was < 10 % for all the variables. Anaemia was defined as Hb concentrations < 110 g/l and < 115 g/l for children aged 6–59 months and 5–11 years, respectively^(22)^. Serum ferritin concentrations < 12 mg/l for children aged < 5 years and < 15 mg/l for those aged ≥ 5 years were used to identify children with Fe deficiency^(22)^ in the absence of signs of inflammation (CRP concentration < 5 mg/l and AGP concentration ≤ 1 g/l). In case of inflammation, ferritin levels were adjusted based on the inflammation stage^(23)^: a correction factor of 0·77 was used for incubation stage (CRP > 5 mg/l & AGP ≤ 1 g/l), 0·53 for early convalescence stage (CRP > 5 mg/l & AGP > 1 g/l) and 0·75 for late convalescence stage (CRP ≤ 5 mg/l and AGP > 1 g/l). Serum retinol concentration < 0·70 µmol/l was used as a cut-off to define low serum retinol^(24)^ as overall there is no issue of severe vitamin A deficiency in Vietnam^(13)^. The prevalence of vitamin D deficiency and insufficiency was determined based on circulating 25-hydroxyvitamin D concentration < 25 nmol/l and from 25 to < 50 nmol/l resp^(25)^. Serum vitamin B_12_ concentration < 150 pmol/l was used as a cut-off to define low serum vitamin B_12_^(26)^. Low serum Zn concentration was defined using the International Zinc Nutrition Consultative Group (IZiNCG) cut-offs by Zn < 65 µg/dl for children < 10 years old and Zn < 70 µg/dl for children ≥ 10 years old^(27)^.

### Dietary intake assessment

Dietary intake was assessed using one-day 24 h dietary recall and food diary. Parents got a record and were requested to write down all foods that the child consumed on the day before data collection was done via 24-h dietary recall. Food diary or record was used to check the food items that children consumed. Different approaches were used for each age group. For children aged 6 months to 9 years, parent-proxy reporting by mother or main caregiver through face-to-face interview was used. For children 10 years and above, parent-proxy reporting by mother or main caregiver combined with child self-reporting was applied. Intake data were collected from midnight to midnight. The food intake data from the 24-h recall were converted to nutrient intakes using Access program developed by the NIN, using the Vietnamese food composition table and compared with Vietnamese recommended nutrient intakes (RNI) and estimated average requirements^(28)^.

Regarding breastmilk consumption, estimating the volume of breastmilk intake for those below 12 months of age was based on a fixed volume set as total daily intake, whereas for those from 12 to 23 months of age, it was a fixed volume per feed^(29)^.

### Statistical analysis

Statistical analysis was performed using RStudio Statistics package version 2022.07.0 with complex samples module. Descriptive analysis was performed and presented as mean and se or presented as percentage. Bivariate analysis was carried out between the parameters of interest using chi square test for categorical outcomes or Student *t* test for continuous outcomes. Throughout the study, a *P* value < 0·05 was considered statistically significant when applying two-sided testing. To produce population estimated, we calculated population-weighted age-specific estimates for each subgroup by followed three steps calculation. In the first step, we calculated the probability of selection of province within the regions. In the second step, the probability of selected cluster was calculated. In the last step, the probability of selection for target in each measurement (anthropometry, biomarker, and dietary intake) was calculated.

## Results

The number of children who participated in the studies is presented below in Table 1. A total of 4001 children participated, with each sex representing approximately 50 % of the total. However, the number of children who were living in rural areas was more than twice as that of children who were living in urban areas, with 2787 and 1214, respectively.

**Table 1:** Number of children who participated in the study by age group, sex and area of residence

	0·5–0·9		1·0–3·9		4·0–6·9		7·0–11·9		Total	
Age (years)	Sample	Estimated population	Sample	Estimated population	Sample	Estimated population	Sample	Estimated population	Sample	Estimated population
Urban										
Boys	47	87 336	190	463 422	124	403 958	255	1 007 846	616	1 962 562
Girls	56	102 473	198	473 121	107	349 967	237	968 475	598	1 894 036
Total	103	189 809	388	936 543	231	753 925	492	1 976 321	1214	3 856 598
Rural										
Boys	128	144 138	452	750 072	282	504 406	542	1 495 857	1404	2 895 473
Girls	118	132 771	439	716 648	276	518 648	550	1 582 721	1383	2 950 788
Total	246	276 909	891	1 466 720	558	1 023 054	1092	3 078 578	2787	5 846 261
Urban and rural										
Boys	175	232 237	642	1 212 732	406	908 365	797	2 504 710	2020	4 858 044
Girls	174	235 244	637	1 189 769	383	868 615	787	2 551 196	1981	4 844 824
Total	349	467 481	1279	2 402 501	789	1 776 980	1584	5 055 906	4001	9 702 868

The anthropometric summary of the children is shown in Table 2. Boys were mostly statistically significant taller and heavier than girls in all age groups (*P* value < 0·05) with some exceptions: height for 4·0 to 11·9 years old. When comparing area of residence, the two youngest age groups did not show any statistically significant differences for all parameters. However, for the older age groups, urban children were heavier, taller and had higher BMI than their rural counterparts.

**Table 2:** Anthropometric characteristics by age group, sex and area of residence

	Urban				Rural				Total					
	Boys		Girls		Boys		Girls		Boys		Girls		General	
	Mean	se	Mean	se	Mean	se	Mean	se	Mean	se	Mean	se	Mean	se
0·5–0·9 years														
Age (years)	0·75	0·03	0·72††	0·02	0·73**	0·02	0·79	0·02	0·74	0·02	0·76	0·01	0·75	0·01
Weight (kg)	8·4	0·2	8·0	0·2	8·7***	0·1	8·0	0·2	8·6***	0·1	8·0	0·1	8·3	0·1
Height (cm)	71·3***	0·7	68·9	0·5	70·4**	0·4	69·2	0·4	70·8***	0·4	69·1	0·3	69·9	0·2
BMI (kg/m2)	16·5††	0·4	16·8	0·2	17·4***	0·2	16·7	0·3	17·1	0·2	16·8	0·2	16·9	0·1
1·0–3·9 years														
Age (years)	2·33††	0·09	2·37†	0·09	2·53	0·07	2·55	0·06	2·45	0·06	2·48	0·05	2·47	0·04
Weight (kg)	12·9***	0·3	11·9	0·3	12·8***	0·2	12·0	0·2	12·8***	0·2	12·0	0·2	12·4	0·1
Height (cm)	88·0	0·9	87·3	0·9	88·7*	0·7	87·3	0·7	88·5*	0·6	87·3	0·5	87·9	0·4
BMI (kg/m2)	16·5†,***	0·2	15·6	0·2	16·1***	0·1	15·6	0·1	16·3***	0·1	15·6	0·1	15·9	0·1
4·0–6·9 years														
Age (years)	5·71	0·12	5·75	0·13	5·76	0·06	5·72	0·07	5·74	0·06	5·74	0·07	5·74	0·05
Weight (kg)	21·7†††	0·7	20·3†	0·9	19·3	0·3	19·0	0·4	20·4*	0·3	19·5	0·4	19·9	0·3
Height (cm)	113·4†	1·1	112·5†	1·1	111·1	0·5	110·6	0·6	112·1	0·6	111·4	0·6	111·7	0·4
BMI (kg/m2)	16·6†††,*	0·3	15·7	0·4	15·5	0·2	15·4	0·2	16·0**	0·2	15·5	0·2	15·7	0·1
7·0–11·9 years														
Age (years)	9·12†	0·08	9·10†	0·09	9·30	0·06	9·28	0·06	9·22	0·05	9·21	0·05	9·22	0·04
Weight (kg)	34·4†††	0·7	32·9†††	0·7	28·6	0·4	27·8	0·4	30·9**	0·4	29·7	0·4	30·3	0·3
Height (cm)	133·2†††	0·6	133·5†††	0·7	129·9	0·5	130·0	0·5	131·2	0·4	131·3	0·4	131·3	0·3
BMI (kg/m^2^)	19·1†††,**	0·3	18·2†††	0·3	16·7**	0·2	16·2	0·2	17·6***	0·2	17·0	0·2	17·3	0·1

SE, standard error; BMI, body mass index.

Mean values were significantly different from girls of each age group based on complex sampling Student *t* test: *P < 0·05; **P < 0·01; ***P < 0·001.

Mean values were significantly different from rural children based on complex sampling Student *t* test: †*P* < 0·05; ††*P* < 0·01; †††*P* < 0·001.

Overall, Vietnamese children, irrespective of sex or area of residence, are presented with a negative mean for most nutritional status z-scores, with a few exceptions (Table 3). Boys had higher Z-scores than girls (*P* value < 0·05) except for HAZ of 1·0–3·9 years, HAZ and WHZ of 4·0–6·9 years, HAZ of 7·0–11·9 years and all scores for 0·5–0·9 years. A relatively similar trend was also seen when comparing between urban and rural, with urban children having higher Z-scores than rural children and the differences were more pronounced (*P* value < 0·5) in the older age groups, except for HAZ of 0·5–0·9 years, WHZ and BAZ of 1–3·9 years and WHZ of 4·0–6·9 years.

**Table 3:** Anthropometric nutritional status indices by age group, sex and area of residence

	Urban				Rural				Total					
	Boys		Girls		Boys		Girls		Boys		Girls		General	
	Mean	se	Mean	se	Mean	se	Mean	se	Mean	se	Mean	se	Mean	se
0·5–0·9 years														
HAZ	−0·27	0·19	−0·27	0·17	−0·53	0·12	−0·58	0·15	−0·43	0·11	−0·45	0·11	−0·44	0·08
WAZ	−0·55	0·22	−0·15	0·16	−0·20	0·10	−0·40	0·20	−0·33	0·11	−0·29	0·13	−0·31	0·09
WHZ	−0·49††,*	0·27	0·06	0·14	0·18	0·11	−0·06	0·18	−0·08	0·14	−0·01	0·12	−0·04	0·09
BAZ	−0·54††,*	0·27	0·01	0·14	0·15	0·11	−0·09	0·18	−0·11	0·14	−0·04	0·12	−0·08	0·09
1·0–3·9 years														
HAZ	−0·51†††	0·09	−0·43†††	0·11	−0·86	0·10	−0·92	0·08	−0·73	0·07	−0·73	0·07	−0·73	0·05
WAZ	−0·06††,*	0·12	−0·33††	0·10	−0·41*	0·08	−0·56	0·08	−0·28**	0·07	−0·47	0·06	−0·37	0·05
WHZ	0·28†,***	0·14	−0·17	0·12	0·05*	0·09	−0·10	0·08	0·14***	0·08	−0·13	0·07	0·00	0·05
BAZ	0·36***	0·14	−0·12	0·12	0·17*	0·10	0·01	0·08	0·24***	0·08	−0·04	0·07	0·10	0·05
4·0–6·9 years														
HAZ	−0·15†††	0·12	−0·21††	0·13	−0·68	0·08	−0·58	0·08	−0·45	0·07	−0·43	0·07	−0·44	0·05
WAZ	0·36†††,*	0·16	−0·15	0·21	−0·46	0·10	−0·44	0·11	−0·10*	0·09	−0·33	0·11	−0·21	0·07
WHZ	−0·04	0·34	−0·72	0·52	0·09	0·21	−0·08	0·25	0·03	0·19	−0·35	0·26	−0·15	0·16
BAZ	0·64†††,**	0·18	−0·09	0·24	−0·05	0·10	−0·13	0·11	0·25***	0·10	−0·11	0·12	0·07	0·08
7·0–11·9 years														
HAZ	0·01†††	0·07	0·04†††	0·09	−0·67	0·07	−0·69	0·07	−0·40	0·05	−0·41	0·06	−0·40	0·04
WAZ	0·79†††,*	0·12	0·47†††	0·12	−0·29*	0·10	−0·55	0·09	0·17***	0·09	−0·14	0·08	0·01	0·06
BAZ	1·07†††,***	0·12	0·63†††	0·11	−0·08**	0·09	−0·31	0·07	0·38***	0·08	0·05	0·06	0·21	0·05

se, standard error; HAZ, height-for-age z score; WAZ, weight-for-age z score; WHZ, weight-for-height z score BAZ, BMI-for-age *z* score.

Mean values were significantly different from girls of each age group based on complex sampling Student *t* test: *P < 0·05; **P < 0·01; ***P < 0·001.

Mean values were significantly different from rural children based on complex sampling Student *t* test: †*P* < 0·05; ††*P* < 0·01; †††*P* < 0·001.

In Table 4, the prevalence of malnutrition per age group, sex and residence is shown. Overall prevalence of stunting was 8·7 % with the highest prevalence in the age group of 1–3·9 years old (12·1 %). In addition, for the total age group, stunting was more prevalent in rural compared with urban children (12·1 % *v*. 3·5 %. *P* < 0·001). Underweight < 5 years old was 5·9 % with again higher prevalence in rural children (7·4 %) compared with their urban counterparts (3·7 %, *P* < 0·001). The opposite trend was seen for overweight and obesity, where urban children had higher prevalence (18·7 % and 16·2 %, respectively) compared with rural children (9·3 % and 6·6 %m respectively, *P* < 0·001, for the total age group). Regarding obesity, more boys (13·9 %) were affected compared with girls (6·9 %, *P* < 0·001). While the prevalence for stunted, wasted and underweight remained relatively similar over the different age groups, prevalence for overweight and obesity was relatively low in the youngest age group (3·2 % and 0·3 %, respectively) compared with the oldest age group (18·1 % and 14·6 %, respectively). The trend was visible in both urban and rural children and for both boys and girls.

**Table 4:** Percentage of children with stunting, wasting, underweight, thinness, overweight and obesity per age group, sex and area of residence

	Urban			Rural			Total		
	Boys	Girls	Total	Boys	Girls	Total	Boys	Girls	Total
0·5–0·9 years									
Stunting	3·9	2·3	3·0†	11·0	9·4	10·2	8·3	6·3	7·3
Underweight	10·7*	0·7†	5·3	3·8	8·2	5·9	6·4	4·9	5·6
Wasted	9·3†	1·5	5·1	2·5	7·9	5·1	5·1	5·1	5·1
Overweight	0·9	2·1	1·6	1·2*	8·0	4·4	1·1*	5·3	3·2
Obesity	0·9	0·0	0·4	0·0	0·6	0·3	0·3	0·3	0·3
1·0–3·9 years									
Stunting	11·5*	5·1†††	8·2†††	15·3	13·7	14·5	13·8*	10·3	12·1
Underweight	2·9	2·6††	2·7†††	6·1	8·5	7·3	4·9	6·2	5·5
Wasted	1·9	4·4	3·2	2·7	2·8	2·8	2·4	3·4	2·9
Overweight	8·5**	2·0	5·3	5·2	2·8	4·0	6·5***	2·5	4·5
Obesity	4·7	2·1†	3·4†	2·5**	0·4	1·4	3·3**	1·1	2·2
4·0–6·9 years									
Stunting	2·9†††	1·1†††	2·1†††	12·2	10·5	11·3	8·2	6·7	7·4
Underweight‡	0·6††,*	13·5	6·6	14·8*	4·4	9·7	8·2	8·4	8·3
Wasted‡	7·3	17·7††	12·1††	4·8	0·0	2·5	6·0	7·9	6·9
Thinness§	0·9**	8·7†	4·6	3·5	2·7	3·1	2·4*	5·1	3·7
Overweight	17·8††	21·0†††	19·3†††	8·3	6·7	7·5	12·4	12·3	12·3
Obesity	17·9†	12·0	15·1†	10·2	8·1	9·1	13·5	9·7	11·6
7·0–11·9 years									
Stunted	1·2†††	2·4†††	1·8†††	11·5	11·2	11·3	7·3	7·9	7·6
Thinness	1·8†††	2·6††	2·2†††	8·3	6·8	7·5	5·7	5·2	5·4
Overweight	26·2†††	26·4†††	26·3†††	13·3	12·5	12·9	18·4	17·8	18·1
Obesity	30·5†††,***	17·3†††	24·0†††	13·3***	4·1	8·6	20·2***	9·1	14·6
0·5–11·9 years									
Stunting	4·1†††	2·8†††	3·5†††	12·5	11·6	12·1	9·2	8·2	8·7
Underweight‡	3·5	3·9	3·7††	6·8	8·0	7·4	5·5	6·3	5·9
Wasted‡	3·8	5·9	4·9	2·9	3·1	3·0	3·3	4·3	3·8
Thinness§	1·1†††,*	2·9	2·0†††	4·9	4·2	4·5	3·4	3·7	3·5
Overweight	19·3†††	18·2†††	18·7†††	9·7	9·0	9·3	13·5	12·5	13·0
Obesity	20·6†††,***	11·7†††	16·2†††	9·4***	3·8	6·6	13·9***	6·9	10·4
0·5–4·9 years									
Stunting	9·1†,*	4·5†††	6·7†††	13·8	12·6	13·2	11·9	9·3	10·6
Underweight	3·5†	3·9†	3·7†††	6·8	8·0	7·4	5·5	6·3	5·9
Wasted	3·8	5·9†	4·9†	2·9	3·1	3·0	3·3	4·3	3·8
Overweight	7·9	4·0	6·0	4·8	3·6	4·2	6·0*	3·8	4·9
Obesity	3·7	1·6	2·6	2·7*	0·9	1·9	3·1**	1·2	2·2

Definition of nutritional status: stunted: height-for-age (HAZ) < –2 sd from the median; underweight (under 5 years only): weight-for-age (WAZ) < –2 sd from the median; wasted (under 5 years only): weight-for-height (WHZ) < –2 sd from the median; thinness (5–11·9 years only): BMI-for-age (BAZ) < –2 sd from the median; overweight: BMI-for-age 2 sd < (BAZ) ≤ 3 sd (< 5 years) and 1 sd < (5–11·9 years) ≤ 2 sd from the median; obese: BMI-for-age (BAZ) > 3 sd (< 5 years) and > 2 sd (5–11·9 years) from the median.

Percentage values were significantly different from girls of each age group complex sampling *χ* ^2^ test: *P < 0·05; **P < 0·01; ***P < 0·001.

Percentage values were significantly different from rural children based on complex sampling *χ* ^2^ test: †*P* < 0·05; ††*P* < 0·01; †††*P* < 0·001.

‡ The data analyses involved children below 5 years old only.

§ The data analyses involved children from 5 years onwards only.

Table 5 shows the results on the micronutrient status of the children. Overall anaemia prevalence was 12·0 % with the highest prevalence found in the youngest children (38·6 % in 0·5–0·9 years old and 15·0 % in the 1–3·9 years old). In addition, rural children were more affected than urban children. Iron deficiency adjusted for inflammation was higher in the 4–6·9 years old age group (8·3 %) compared with the 7–11·9 years old (2·5 %). Around 6·2 % of the children were affected with low serum retinol, while 8·0 % had vitamin B_12_ deficiency. Vitamin D insufficiency was 30·4 % while less than 1 % had vitamin D deficiency. The prevalence was higher in urban children compared with rural (41·3 % *v*. 23·3 %, respectively, *P* < 0·001), and girls were more often affected than boys (38·6 % *v*. 22·5 % respectively, *P* < 0·001). Prevalence of low serum Zn was very high with 60·8 % of the children affected. However, there were no differences between sex and residence, and prevalence over the age groups was similar.

**Table 5:** Prevalence of anemia, iron deficiency iron deficiency adjusted for inflammation, low serum retinol, vitamin D insufficiency and deficiency, vitamin B_12_ deficiency and low serum zinc by age group, sex and area of residence

	Urban			Rural			Total		
	Boys	Girls	Total	Boys	Girls	Total	Boys	Girls	Total
0·5–0·9 years									
Anaemia	23·5†	36·8	29·3†	50·2	47·1	48·5	35·0	42·4	38·6
1·0–3·9 years									
Anaemia	6·6†	14·0	10·0†	17·2	19·3	18·1	13·2	17·2	15·0
4·0–6·9 years									
Anaemia	7·6	9·8	8·6	9·0	13·1	10·9	8·3	11·8	9·9
Fe deficiency	10·8	1·4†	6·7	7·4	10·3	8·8	8·9	6·7	7·9
Iron deficiency adj	10·8	1·4†	6·7	8·6	10·0	9·4	9·6	6·7	8·3
Low serum retinol	7·0	4·7	6·0	4·8	8·3	6·4	5·8	6·9	6·3
Vitamin D insufficiency	21·2	30·4	25·2	12·2*	22·6	17·1	16·1*	25·8	20·5
Vitamin B_12_ deficiency	5·7	8·0	6·7	6·7	2·7	4·8	6·3	4·8	5·6
Low serum Z	62·8	54·8	59·3	61·3	62·7	61·9	62·0	59·5	60·8
7·0–11·9 years									
Anaemia	5·2	5·1	5·1†	7·0	11·1	9·2	6·3	9·0	7·6
Fe deficiency	1·5	6·3†	3·6	2·9*	0·2	1·5	2·3	2·3	2·3
Fe deficiency adj	1·7	6·3†	3·8	3·2**	0·2	1·6	2·6	2·3	2·5
Low serum retinol	5·0	2·6	3·9†	9·7	5·6	7·5	7·7	4·6	6·1
Vitamin D insufficiency	34·7†††,***	62·0†††,***	47·1†††	17·5	32·0	25·2	24·8***	42·4	33·6
Vitamin D deficiency	0·2	3·7†	1·8	0·0	0·7	0·4	0·1*	1·8	0·9
Vitamin B_12_ deficiency	5·6†	2·6††	4·2†††	13·0	10·4	11·6	9·9	7·7	8·8
Low serum Zn	60·2	59·7	60·0	65·4	57·6	61·2	63·2	58·3	60·8
0·5–11·9 years									
Anaemia	7·7†	10·8	9·1††	12·4	15·4	13·9	10·4*	13·7	12·0
Fe deficiency	4·0	5·0	4·4	4·0	2·3	3·2	4·0	3·3	3·7
Fe deficiency adj	4·2	5·0	4·6	4·6	2·3	3·4	4·4	3·3	3·9
Low serum retinol	5·5	3·1	4·5	8·5	6·2	7·3	7·2	5·1	6·2
Vitamin D insufficiency	31·1†††,***	53·8†††	41·3†††	16·1***	30·0	23·3	22·5***	38·6	30·4
Vitamin D deficiency	0·1*	2·7†	1·3	0·0	0·6	0·3	0·1*	1·4	0·7
Vitamin B_12_ deficiency	5·6	4·0	4·9	11·4	8·8	10·0	8·9	7·1	8·0
Low serum Zn	60·9	58·4	59·8	64·3	58·7	61·4	62·9	58·6	60·8
0·5–4·9 years									
Anaemia	13·6	17·7	15·5†	21·0	25·1	22·9	17·9	22·0	19·8

Prevalence of anaemia, Hb level: < 110 g/l (children < 5 years), < 115 g/l (5–11·9 years).

Iron deficiency, in the absence of inflammation, ferritin level: < 12 µg/l (children < 5 years), < 15 µg/l (children ≥ 5 years); if inflammation was present correction factors were applied to the ferritin levels by multiplying with 0·77 for the incubation stage (C-reactive protein (CRP) > 5 mg/l and alpha-glycoprotein (AGP) ≤ 1 g/l), 0·53 for the early convalescence stage (CRP > 5 mg/l and AGP > 1 g/l) and 0·75 for the late convalescence stage (CRP ≤ 5 mg/l and AGP > 1 g/l).

Low serum retinol, retinol level: < 0·7 µmol/l.

Vitamin D insufficiency, 25-hydroxyvitamin D level between 25–50 nmol/l; vitamin D deficiency, 25-hydroxyvitamin D level < 25 nmol/l.

Vitamin B_12_ deficiency, vitamin B_12_ level < 150 pmol/l.

Low serum Zn, Zn level: < 65 µg/dl (children < 10 years, non-fasting), < 70 µg/dl (girls ≥ 10 years, morning-fasting) and < 74 µg/dl (boys ≥ 10 years, morning-fasting).

Percentage values were significantly different from girls of each age group based on complex sampling *χ* ^2^ test: *P < 0·05; **P < 0·01; ***P < 0·001.

Percentage values were significantly different from rural children based on complex sampling *χ* ^2^ test: †*P* < 0·05; ††*P* < 0·01; †††*P* < 0·001.

Table 6 shows the nutrient intake as calculated from the 24-h recall. In general, urban children had a higher nutrient intake compared with rural children. Differences in nutrient intake between boys and girls were mainly found in the oldest age group, 7–11·9 years old, where boys had higher intakes than girls.

**Table 6:** Nutrient intake by age group, sex and area of residence

	Urban				Rural				Total					
	Boys		Girls		Boys		Girls		Boys		Girls		General	
	Mean	se	Mean	se	Mean	se	Mean	se	Mean	se	Mean	se	Mean	se
0·5–0·9 years														
Energy (kcal)	703·3†	39·2	641·4	25·5	607·1	19·5	580·0	18·30	643·9	18·4	606·4	15·0	625·0	11·9
Protein (g)	23·0	1·2	22·8	1·1	22·0	0·9	20·8	0·9	22·4	0·8	21·7	0·7	22·0	0·5
CHO (g)	97·5*,†	6·9	81·2	3·7	77·1	3·3	74·4	2·6	84·9	3·2	77·3	2·1	81·1	1·9
Fat (g)	23·8	1·4	24·7†	1·1	22·9	0·7	21·7	0·8	23·3	0·6	23·0	0·6	23·1	0·5
Ca (mg)	400·6	29·9	436·0†	29·5	359·6	17·5	350·6	16·7	375·3	15·2	387·3	15·5	381·4	10·9
Fe (mg)	5·6	0·5	5·5††	0·4	4·6	0·3	4·0	0·2	5·0	0·3	4·6	0·2	4·8	0·2
Zn (mg)	5·1	0·3	4·8††	0·2	4·5	0·2	4·1	0·1	4·7	0·1	4·4	0·1	4·5	0·1
Vit A (µg-RAE)	592·9	41·3	611·3	37·5	591·2	23·3	596·6	39·5	591·8	20·5	603·0	28·2	597·4	17·4
Vit B_12_ (µg)	1·2	0·1	1·2	0·1	1·3	0·1	1·2	0·1	1·2	0·09	1·2	0·08	1·2	0·06
Vit C (mg)	57·5	4·6	56·5††	3·5	47·9	2·3	43·8	2·0	51·6	2·2	49·3	1·9	50·4	1·4
Vit D (µg)	4·5	0·6	4·2	0·5	3·6	0·4	4·0	0·5	3·9	0·3	4·1	0·3	4·0	0·2
1·0–3·9 years														
Energy (kcal)	976·7†††	25·2	956·3†††	22·1	868·2	13·2	859·7	13·1	909·5	12·4	898·4	11·7	904·0	8·5
Protein (g)	42·5†††	1·2	40·7†††	1·0	35·9	0·6	35·6	0·7	38·4	0·6	37·7	0·6	38·0	0·4
CHO (g)	134·4†	3·5	132·7	3·5	125·8	2·2	125·0	2·1	129·0	1·9	128·1	1·8	128·6	1·3
Fat (g)	29·3†††	1·0	28·8†††	0·9	24·5	0·5	24·0	0·5	26·3	0·5	25·9	0·5	26·1	0·3
Ca (mg)	587·3†††	18·6	551·5†††	17·7	450·8*	10·5	482·0	10·7	502·8	9·7	509·8	9·4	506·3	6·75
Fe (mg)	7·3**,†††	0·2	6·4†††	0·2	5·4	0·1	5·4	0·1	6·1	0·1	5·8	0·1	6·0	0·08
Zn (mg)	6·4†††	0·2	5·9†††	0·1	5·3	0·09	5·2	0·09	5·7*	0·08	5·5	0·08	5·6	0·06
Vit A (µg-RAE)	459·5	21·2	448·0	21·0	429·2	19·3	431·7	18·1	440·7	14·6	438·2	13·8	439·5	10·0
Vit B_12_ (µg)	2·3	0·1	2·3	0·1	2·1	0·1	2·1	0·08	2·2	0·08	2·1	0·07	2·2	0·05
Vit C (mg)	43·6	3·1	44·8	3·2	38·6**	2·0	49·5	3·0	40·5*	1·7	47·6	2·3	44·0	1·4
Vit D (µg)	5·8	0·3	5·3	0·3	5·8	0·3	5·6	0·3	5·8	0·2	5·5	0·2	5·6	0·2
4·0–6·9 years														
Energy (kcal)	1282·6†††	34·8	1264·3†††	43·7	1096·8	21·0	1082·8	23·1	1179·1	18·9	1157·0	21·6	1168·2	14·3
Protein (g)	59·8†††	1·8	58·4†††	2·6	46·6	1·1	45·3	1·1	52·4	1·0	50·6	1·2	51·6	0·8
CHO (g)	183·5††	5·4	175·9†	4·9	163·4	3·2	163·1	3·6	172·3	2·8	168·3	2·9	170·4	2·0
Fat (g)	34·4†††	1·3	36·2†††	2·2	28·5	0·9	27·7	0·8	31·1	0·8	31·1	1·0	31·1	0·6
Ca (mg)	548·5†††	26·1	493·8†††	23·4	373·8	14·4	379·7	13·5	451·1	13·8	426·3	12·2	438·9	9·2
Fe (mg)	8·0†††	0·3	8·4†††	0·4	6·4	0·2	6·1	0·2	7·1	0·2	7·1	0·2	7·1	0·1
Zn (mg)	7·3†††	0·2	7·4†††	0·4	6·0	0·1	5·8	0·1	6·6	0·1	6·4	0·2	6·5	0·1
Vit A (µg-RAE)	394·2	23·9	379·7	27·9	342·7	18·9	347·9	23·6	365·5	14·7	360·9	18·1	363·2	11·6
Vit B_12_ (µg)	3·3††	0·4	2·9††	0·3	2·1	0·1	2·0	0·1	2·6	0·2	2·3	0·1	2·5	0·1
Vit C (mg)	50·8†	6·8	47·0†	5·6	33·5	2·7	33·7	2·5	41·1	3·0	39·2	2·5	40·2	2·0
Vit D (µg)	5·2	0·5	4·5	0·4	4·6	0·3	4·1	0·3	4·9	0·3	4·3	0·2	4·6	0·2
7·0–11·9 years														
Energy (kcal)	1513·9***,†††	33·5	1299·3†††	24·9	1304·0***	19·0	1178·1	18·6	1389·2***	17·5	1223·3	15·0	1305·5	11·7
Protein (g)	68·4**,†††	1·5	61·8†††	1·7	55·7***	1·1	49·0	10·9	60·9***	0·9	53·8	0·9	57·3	0·6
CHO (g)	223·9***,†††	5·7	186·7	3·8	199·8***	3·0	183·8	3·0	209·6***	2·8	184·9	2·4	197·1	1·9
Fat (g)	38·4*,†††	1·4	34·1†††	1·2	31·3**	0·9	27·4	0·8	34·2***	0·8	29·9	0·7	32·0	0·5
Ca (mg)	474·4†††	20·8	446·2†††	18·9	332·2**	9·8	292·9	8·8	389·9**	10·1	350·0	9·0	369·8	6·8
Fe (mg)	8·9†	0·2	8·5†††	0·2	8·3***	0·2	7·4	0·1	8·6***	0·10	7·8	0·1	8·2	0·09
Zn (mg)	8·4**,†††	0·2	7·6†††	0·2	7·3***	0·1	6·6	0·1	7·8***	0·1	7·0	0·1	7·4	0·08
Vit A (µg-RAE)	339·6	17·3	347·6†	21·2	379·7	17·5	414·0	22·9	363·4	12·7	389·2	16·8	376·4	10·55
Vit B_12_ (µg)	2·7*,†	0·2	4·7††	1·0	2·1	0·2	1·7	0·1	2·3	0·1	2·9	0·3	2·6	0·2
Vit C (mg)	53·8†	4·1	52·2	4·1	44·1	2·4	48·2	2·7	48·0	2·1	49·7	2·3	48·9	1·6
Vit D (µg)	4·4	0·3	4·2	0·5	3·8	0·3	3·2	0·2	4·0	0·2	3·5	0·2	3·8	0·2

Mean values were significantly different from girls of each age group based on complex sampling Student *t* test: *P < 0·05; **P < 0·01; ***P < 0·001.

Mean values were significantly different from rural children based on complex sampling Student *t* test: †*P* < 0·05; ††*P* < 0·01; †††*P* < 0·001.

Tables 7 and online Supplementary Table 1 show the prevalence of children not meeting Vietnamese RNI and estimated average requirements, respectively. Overall, urban children showed lower prevalence of not meeting RNI for all nutrients, except, vitamin A and vitamin D, where there was no difference between residences. Not meeting the recommendations for energy intake was a concern in all age groups. Almost 60 % of the children 0·5–0·9 years old were not meeting energy intake recommendations, while this applied for almost 90 % of the oldest children. Nutrients of concern, where > 50 % of the children were not meeting RNI, were Ca, Fe and vitamins A, C and D. Regarding Ca, around 60 % of the children between 0·5 and 3·9 years old were not meeting the RNI, while in the older age groups it was 79·4 % and 92·6 % for the 4–6·9- and 7–11·9-years old children, respectively. Intake of Fe is a concern in mainly the youngest children (86·4 %) and oldest children (68·5 %). In contrast to age group-specific results for Ca and Fe, prevalence of not meeting recommended intake of vitamin D was > 90 % across all age groups.

**Table 7:** Percentage of children not meeting EER for energy and RNI for the other nutrients per age group, sex and area of residence

	Urban			Rural			Total		
	Boys	Girls	Total	Boys	Girls	Total	Boys	Girls	Total
0·5–0·9 years									
Energy	50·6	49·2	49·8††	65·0	62·8	63·9	59·5	56·9	58·2
Protein	36·2	35·5††	35·8††	45·6	56·5	50·9	42·0	47·4	44·7
Ca	62·5	53·3	57·6	66·3	64·5	65·4	64·9	59·6	62·2
Fe	84·2	83·9	84·1	87·2	89·0	88·1	86·1	86·8	86·4
Zn	39·5	36·7†	38·0††	54·0	54·6	54·3	48·5	46·9	47·7
Vit A	25·9	22·0	23·8	21·1	31·4	26·1	22·9	27·4	25·1
Vit B_12_	16·4	19·3†	17·9††	28·4	33·2	30·7	23·8	27·2	25·5
Vit C	37·1	23·5†††	29·8††	42·0	49·2	45·5	40·1	38·2	39·1
Vit D	93·0	94·7	93·9	94·3	93·1	93·7	93·8	93·8	93·8
1·0–3·9 years									
Energy	69·6†††	60·1††	64·8†††	81·3**	72·5	77·0	76·8***	67·5	72·2
Protein	6·9†	4·7†	5·8††	12·4	9·5	10·9	10·3	7·6	8·9
Ca	44·2*,†††	54·6	49·5†††	69·0*	62·4	65·8	59·6	59·3	59·4
Fe	30·7†††	31·1†††	30·9†††	55·1	49·4	52·4	45·8	42·1	44·0
Zn	19·6††	19·5†††	19·6†††	31·5*	38·7	35·0	27·0	31·0	29·0
Vit A	55·3	46·3	50·7†	61·4*	53·8	57·7	59·1**	50·8	55·0
Vit B_12_	13·8†	15·4	14·6†	21·5	18·9	20·3	18·6	17·5	18·1
Vit C	52·0†	55·6	53·8†	61·2	60·4	60·8	57·7	58·5	58·1
Vit D	96·1	96·6	96·3†	93·7	93·6	93·7	94·6	94·8	94·7
4·0–6·9 years									
Energy	74·3	63·5††	69·2††	81·4	76·0	78·7	78·2*	70·9	74·6
Protein	1·3*,†††	7·1††	4·0†††	19·5	19·0	19·2	11·5	14·1	12·8
Ca	63·6†††	73·9†††	68·4†††	86·6	88·4	87·6	76·4*	82·5	79·4
Iron	35·1†††	25·8†††	30·7†††	57·1	55·8	56·5	47·3	43·6	45·5
Zn	13·1†††	19·5†††	16·1†††	36·7*	45·6	41·2	26·3**	34·9	30·5
Vit A	72·0	65·2	68·8	76·7	71·0	73·8	74·6	68·6	71·7
Vit B_12_	11·8†††	17·1†††	14·3†††	37·3	33·4	35·3	26·0	26·8	26·4
Vit C	69·8	71·6	70·7	77·1	74·6	75·8	73·9	73·4	73·6
Vit D	96·1	94·3	95·3	93·3	95·4	94·3	94·5	95·0	94·7
7·0–11·9 years									
Energy	78·4***,†††	90·1	84·1†††	89·2	91·1	90·2	84·8***	90·7	87·8
Protein	11·2*,†††	18·1†††	14·5†††	29·0**	38·4	33·9	21·7***	30·8	26·3
Ca	84·1*,†††	90·5†††	87·2†††	95·0	96·9	96·0	90·6**	94·5	92·6
Iron	56·5*,†††	67·0†	61·6†††	71·1	74·5	72·8	65·2**	71·7	68·5
Zn	29·5†††	33·7†††	31·5†††	47·6	47·5	47·5	40·3	42·3	41·3
Vit A	80·6	80·1	80·3†	75·7	74·9	75·3	77·7	76·9	77·3
Vit B_12_	34·9†††	37·1†††	36·0†††	58·4	63·4	61·0	48·9	53·6	51·3
Vit C	74·3	75·4	74·8	79·0	74·3	76·6	77·1	74·7	75·9
Vit D	95·0	95·0	95·0	94·4	96·5	95·5	94·7	95·9	95·3
0·5–11·9 years									
Energy	74·3†††	75·3†††	74·8†††	84·6	82·7	83·7	80·4	79·8	80·1
Protein	9·3*,†††	13·6†††	11·4†††	23·8**	28·9	26·4	17·9***	22·9	20·4
Ca	69·6**,†††	76·3†††	72·9†††	85·4	85·7	85·5	79·0*	82·0	80·5
Fe	47·3†††	51·1†††	49·1†††	65·3	65·8	65·6	58·0	60·1	59·0
Zn	24·3†††	27·6†††	25·9†††	41·8	45·3	43·6	34·7*	38·4	36·6
Vit A	70·5	65·7	68·1	69·5	67·2	68·4	69·9*	66·6	68·3
Vit B_12_	24·4†††	26·9†††	25·6†††	43·6	46·1	44·9	35·8	38·7	37·2
Vit C	66·5††	66·9	66·7††	72·2	69·9	71·0	69·9	68·7	69·3
Vit D	95·4	95·2	95·3	94·0	95·5	94·8	94·6	95·4	95·0

Percentage values were significantly different from girls of each age group complex sampling *χ* ^2^ test: *P < 0·05; **P < 0·01; ***P < 0·001.

Percentage values were significantly different from rural children based on complex sampling *χ* ^2^ test: †*P* < 0·05; ††*P* < 0·01; †††*P* < 0·001.

## Discussion

SEANUTS II Vietnam aims to provide a nationally representative overview of the current nutritional and health status of children between 0·5 and 11·9 years old in both urban and rural areas of Vietnam. The results showed that a triple burden of malnutrition is still clearly a concern for Vietnamese children.

SEANUTS II Vietnam showed that undernutrition remains a medium level of public health burden: 10·6 % of children < 5 years old were affected by stunting, while 5·9 % were underweight. These percentages are improvements compared with the SEANUTS I conducted 10 years earlier where 14 % was stunted and 8·6 % underweight^(11)^. Also the GNS from 2000, 2010 and 2020^(9,13,30)^ showed that Vietnam has been working towards its objective of reducing stunting in children: the prevalence of stunting went down from 29·3 % in 2010 to 19·6 % in 2020, while the prevalence of underweight decreased from 17·5 % in 2010 to 11·5 % in 2020. However, undernutrition rates as measured in SEANUTS II are lower than reported by GNS 2020. A possible reason for this difference could be because SEANUTS II’s sampling strategy had a lower coverage of the minor ethnic groups in remote areas from rural Central Highland and North Mountainous areas, which are known to be more affected by undernutrition due to logistics and financial reasons^(14)^.

Prevalence of stunting was higher in rural children compared with urban children (13·2 % *v*. 6·7 %, respectively, in children aged 0·5–4·9 years). Almost two-thirds of Vietnam’s population live in rural areas^(17)^. These areas, especially in the Northern region and Central Highland regions, are characterised by rugged upland terrain, poor infrastructure and low cultivation of agricultural production, leading to high prevalence of household poverty^(31)^. There is limited access to nutritious foods and healthcare services. Although substantial progress has been made in implementing programmes to prevent and combat undernutrition in Vietnamese children, more focus is required for comprehensive communication strategies to reach mothers and children in rural and mountainous areas^(32)^.

Factors involved in undernutrition could be living standard of the household^(33)^ and the educational level of the families, especially the mothers^(34)^ low access to nutritious foods and low power status of women to provide good foods for their children^(35)^. Nutritional improvement is not only important in the first 1000 d of life but should also be an ongoing process, in which the school years also play an essential and equally important role as prep-school age. To improve the stature and physical strength of Vietnamese children, the implementation of school health interventions plays an important role. Recognising this fact, in 2022, Decision 1768 issued by the Ministry of Health specifically identified improving the quality of school meals in poor areas as one intervention within the National Target Program for sustainable poverty reduction aiming to reduce stunting among school children aged 5–16 years to below 34 % by 2025^(36)^.

As with other developing countries, the rise in overweight and obesity is a major public health issues and an important focus of Vietnam’s strategy against malnutrition. SEANUTS II Vietnam showed 23·4 % of the children between 0·5 and 11·9 years old to be overweight or obese. In addition, more urban children are affected (34·9 %) compared with rural (15·9 %). Thus, together with stunting and underweight, Vietnam is facing a double burden of malnutrition.

Studies over the years showed an increase in the prevalence of overnutrition. The GNS in 2000^(9)^ reported 0·62 % of overweight and obesity in children under 5 years of age. The GNS 2010^(13)^ reported 5·6 % of children < 5 years and about 35 % between 5 and 11 years old were overweight/obese in urban areas, while among rural children, these values were about 4 % and 10 %, respectively. Data from SEANUTS I (2010) showed that almost 29 % of the urban children were either overweight or obese compared with 5·6 % of the rural children^(11)^.

Factors that have been indicated to be involved in the development of overweight/ obesity are low physical activity, changes in dietary patterns, with increased consumption of highly processed food, refined carbohydrates or added sugars and fats and increased exposure to mass media shifting children toward unhealthy food choices^(35)^. Future intervention programs in Vietnam need to target prevention of children developing overweight and obesity.

SEANUTS II Vietnam showed that prevalence of anaemia varied between 38·6 % in the youngest children aged 0·5–0·9 years old and 7·6 % in the children 7–11·9 years old with a higher prevalence in rural children compared with urban children. High prevalence of anaemia in the younger children could be due to Fe, folate or vitamin B_12_ deficiency^(37)^. However, we did not measure these biomarkers in children < 4 years old as SEANUTS II implemented venipuncture only in children from 4 years onwards.

Prevalence of vitamin B_12_ deficiency in SEANUTS II Vietnam was 8·0 % irrespective of sex or residence. Very few Asian countries have national data on vitamin B_12_ status in children^(38)^. To our knowledge, SEANUTS II Vietnam is the first study with data on the nationwide prevalence of vitamin B_12_ deficiency in school children in Vietnam. The data are in agreement with an earlier study conducted in 2009 among women of reproductive age from twenty-five provinces in Vietnam showing 12 % of vitamin B_12_ deficiency, with an additional 4 % having a marginal status, i.e. a plasma vitamin B_12_ concentration between 148 and 220 pmol/l^(38)^. A diet that includes meat, fish and dairy products usually provides sufficient vitamin B_12_. First data from SEANUTS II Vietnam showed that < 40 % of the children do not meet RNI for vitamin B_12_ (see Table 7) Further in-depth analysis could provide further insights in the association of vitamin B_12_ intake and status.

More than 60 % of the children between 4 and 11·9 years old had low serum Zn concentration, with no differences between sex, residence or age group. Zn is involved in metabolic activities and plays a critical role in growth and development of children^(39)^. Low serum Zn concentration is associated with suboptimal linear growth and with impaired immune function, leading to recurrent infections. Our findings in SEANUTS II Vietnam are in line with the data from the GNS 2020, which reported 58 % in children of 6–59 months^(30)^. A review including twenty-five low- and middle-income countries described that twenty-three reported > 20 % of low Zn concentration for at least one physiological group such as children, adolescents, men or women age^(40)^. Data from Cambodia from 2014 also found that > 60 % of women of reproductive age and children < 5 years old had low serum Zn concentrations National Institute of Statistics^(41)^. Two studies from Thailand published in 2006 showed that 27·7 % of infants and 57·0 % of school aged children had low serum Zn concentration^(42,43)^. In contrast, children in SEANUTS II Thailand had a much lower prevalence of low serum Zn concentration of 3·7 %^(44)^. Reasons for the high prevalence of low serum zinc concentration in Vietnamese children could be a less diversified diet and lack of foods rich in Zn such as seafood and animal-sourced foods or persistent infectious diseases. However, > 50 % of the children in SEANUTS II Vietnam had sufficient zinc intake compared with Vietnamese RNI (see Table 7), which could indicate Zn absorption is not optimal. Although there has been a decline in the prevalence of low serum Zn concentration compared with the results of GNS 2010 (81·5 % in children 6–59 months old)^(13)^, the results from SEANUTS II advocate that more attention to improve Zn status is needed.

SEANUTS II Vietnam showed a prevalence of 6·2 % of low serum retinol in children from 4 to 11 years old with no differences between sex and residence. However, one in four children had borderline vitamin A levels (serum retinol between 0·7 and 1·05 µmol/l, data not shown). Vitamin A is important for normal functioning of the visual system, growth, epithelial cellular integrity, immune function and reproduction. Low serum retinol can slow down the growth rate and also increase the risk of infectious diseases^(45)^. The GNS 2020 reported 9·2 % of low serum retinol in 6–59 months old children^(30)^. In SEANUTS I (2011), the prevalence of low serum retinol in 6–11·9 years old children was 5·8–9·7 %, whereas about half of the children (48·9 %) have borderline vitamin A levels^(11)^. The key risk factors for low serum retinol are a diet low in sources of vitamin A (i.e. eggs, fruits and vegetables), poor nutritional status and a high rate of infections, in particular, measles and diarrhoeal diseases^(46)^. Although the vitamin A supplementation program has been implemented successfully and the overall prevalence of low serum retinol is lower compared with the burden of anaemia and low Zn concentrations^(47)^, it is still ranked as a mild level of public health problem, and it has not significantly reduced over last 10 years. Therefore, efforts to prevent low serum retinol must be maintained. Treating low serum retinol through vitamin A supplementation has been the focus of universal efforts. However, a study in Philippines showed that the effect of high-dose vitamin A capsules only last between 2 and 3 months with no effect after 6 months^(48)^. This would suggest that other complementary types of intervention, such as food fortification or food diversification, should be planned and evaluated.

Regarding vitamin D insufficiency/ deficiency, 31·1 % of the Vietnamese children between 4 and 11·9 years old were affected. Older children and urban children showed higher percentages. Vitamin D is important for bone health and growth and plays a role in the immune system^(49,50)^. Vitamin D insufficiency is emerging in recent years even though Vietnam is a tropical country spreading from 8° to 23° North latitude with many daily hours of sun exposure. SEANUTS II showed lower rates for vitamin D insufficiency compared with SEANUTS I 41·3 % *v*. 52·7 % for urban and 23·3 % *v*. 48·1 % for rural children, respectively^(11)^. Vitamin D intake data show that >90 % of the children do not meet RNI for vitamin D. Other factors that could be associated with these high rates are full schedules of indoor school days, lack of outdoor physical activities under sunlight and air pollution, particularly in the urban areas. Changes of school curriculum, including breaks and physical education outside, could help in lowering vitamin D insufficiency in children.

SEANUTS II Vietnam showed that rural children had lower intakes of macro- and micronutrient intakes and were less able to meet the RNIs. Similar trends were found in SEANUTS I Vietnam in which urban children also has the higher dietary intake of energy, protein, carbohydrate, fat, iron, vitamin A, vitamin B1 (except in the age group 0·5–1·9 years) and vitamin C (except in the age group 2·0–4·9 years) than rural children^(11)^. However, there seemed to be a slight improvement as percentages not meeting RNI is lower for protein, Fe and vitamin A for SEANUTS II Vietnam, although these results should be interpreted with caution as we conducted a 24 h recall over 1 d only.

Regarding energy intake, almost 60 % of the 0·5–0·9-year-old children did not meet energy intake recommendations, while this percentage was almost 90 % of the children aged 7–11·9 years old. Of these older children not meeting the energy recommendations, around 60 % had an energy intake of < 70 % of the recommendations (data not shown). Most children were meeting recommendations for protein intakes. For further analysis, it would be of interest to study protein quality, i.e. animal *v*. plant protein. For micronutrients, Ca, Fe and vitamins A, C and D are of concern. It would be of interest to study if there are any associations between nutrient intakes and nutritional status. Also, more in-depth analysis of the diet quality and the respective food items/ food groups consumed will provide information to further develop programs to improve the nutrient intake of Vietnamese children.

The strengths of the SEANUTS II Vietnam are the nationally representative availability of anthropometric, biochemical and dietary intake data for children of 0·5–11·9 years old from Vietnam. In addition, information on lifestyle behaviours like physical activity, sedentary behaviour and sleep was collected as well as information on food security and dietary habits. All this information together will provide a comprehensive and holistic data set for further analysis.

A limitation from SEANUTS II Vietnam is that the nutrient intake data are derived from a single 24-h dietary recall, so it is not representing a habitual diet. The study design is cross-sectional, which implies we cannot make any causal inference. Furthermore, there is no information on biochemical data for children < 4 years old, except for Hb. Lastly, data collection was conducted from September 2020 to April 2021, the time that COVID-19 pandemic occurred which could have affected the dietary intake and prevalence of overweight/obesity in children.

In conclusion, Vietnam is still facing a triple burden of malnutrition: undernutrition, overnutrition and micronutrient deficiencies co-exists in the country. Further in-depth analyses of the food consumption data, food habits, physical activity in relation to nutritional status including biochemical parameters are essential to make interpretations and recommendations.

The results of SEAUTS II Vietnam are useful for policymakers to develop effective strategic plans and actions to improve nutritional status and contributing to reduce disease burden of Vietnamese children, for example, by strengthening the nutritional education and communication at schools and the community and implementing good-quality school meal programs. Strategies for improving the nutritional status of children should focus on prevention and control of both undernutrition and overweight and obesity as well as micronutrient deficiencies such as vitamin D insufficiency and low serum Zn.

## Supporting information

Tran et al. supplementary material Tran et al. supplementary material

## SEANUTS II Vietnam Study Group

The SEANUTS II Vietnam Study Group comprises of the following: National Institute of Nutrition: Nga Thuy Tran, Van Khanh Tran, Duong Thanh Tran. Tu Song Nguyen, Phuong Thi Lan Nguyen, Tu Tran Ngoc Nguyen, Anh The Nguyen, Hanh Thi Dang, An Vinh Pham, Anh Thi Van Nguyen, Bac Huu Nguyen, Tung Van Thanh Le, Ngan Thi Pham, Thoan Dieu Nguyen, Trang Thi Huyen Nguyen, Son Duy Nguyen, Ha Thu Nguyen, Phuong Thi Mai Tuan, Hoa Anh Le, Trung Duc Le. FrieslandCampina: Ilse Khouw, Swee Ai Ng, Ye Sun, Panam Parikh, Gerard Wong, Yi Xiu Ng, Huong Bui Thi Thu (FrieslandCampina Vietnam).

## Supplementary material

For supplementary material accompanying this paper visit https://doi.org/10.1017/S1368980024001186
