# Supplementary material for: Triple burden of malnutrition among Vietnamese 0·5–11-year-old children in 2020–2021: results of SEANUTS II Vietnam
Source: Public Health Nutr. 2024 May 24;27(1):e259. doi: 10.1017/S1368980024001186 (PMC11705026; doi:10.1017/S1368980024001186)
Supplement: Tran et al. supplementary material [file S1368980024001186sup001.docx]

Supplementary Table 1 Percentage of children not meeting EAR per age group^1^, sex, and area of residence

|  | **Urban** | | | **Rural** | | | **Total** | | |
| --- | --- | --- | --- | --- | --- | --- | --- | --- | --- |
|  | **Boys** | **Girls** | **Total** | **Boys** | **Girls** | **Total** | **Boys** | **Girls** | **Total** |
| **1.0-3.9 years** | | | | | | | | | |
| Vit A | 32.1††† | 28.5 | 30.3††† | 46.2*** | 34.9 | 40.7 | 40.8** | 32.3 | 36.6 |
| Vit B12 | 8.1†† | 12.3 | 10.2†† | 15.5 | 15.8 | 15.6 | 12.7 | 14.4 | 13.5 |
| Vit C | 44.0†† | 49.2 | 46.6†† | 57.5 | 51.5 | 54.5 | 52.4 | 50.5 | 51.5 |
| **4.0-6.9 years** | | | | | | | | | |
| Vit A | 48.4† | 46.3† | 47.4†† | 59.3 | 59.5 | 59.4 | 54.5 | 54.1 | 54.3 |
| Vit B12 | 7.7††† | 10.3††† | 8.9††† | 33.0 | 29.8 | 31.4 | 21.8 | 21.8 | 21.8 |
| Vit C | 61.0†† | 69.7 | 65.1 | 74.0 | 69.3 | 71.6 | 68.3 | 69.4 | 68.8 |
| **7.0-11.9 years** | | | | | | | | | |
| Vit A | 63.3 | 68.0 | 65.5 | 64.8 | 65.5 | 65.2 | 64.2 | 66.4 | 65.3 |
| Vit B12 | 25.0††† | 29.1††† | 27.0††† | 50.2** | 58.4 | 54.4 | 40.0** | 47.5 | 43.8 |
| Vit C | 70.7 | 67.4 | 69.1† | 76.2 | 72.7 | 74.4 | 74.0 | 70.7 | 72.3 |
| **0.5-11.9 years** | | | | | | | | | |
| Vit A | 52.4†† | 53.1 | 52.8†† | 58.7 | 56.7 | 57.7 | 56.1 | 55.3 | 55.7 |
| Vit B12 | 17.2††† | 20.9††† | 19.0††† | 37.5* | 42.5 | 40.0 | 29.2** | 34.1 | 31.7 |
| Vit C | 62.0††† | 63.0 | 62.5††† | 70.7* | 66.7 | 68.7 | 67.2 | 65.3 | 66.2 |

^1^EAR values are not available for the youngest age group of 0.5-0.9 years.

Percentage values were significantly different from girls of each age group complex sampling Chi-square test: **p*<0.05, ***p*<0.01, ****p*<0.001.

Percentage values were significantly different from rural children based on complex sampling Chi-square test: †*p*<0.05, ††*p*<0.01, †††*p*<0.001.
